# Data-driven FMEA approach for hazard identification and risk evaluation in digital health

**DOI:** 10.1038/s41598-025-11929-4

**Published:** 2025-07-23

**Authors:** Hafiz Muhammad Waseem, Saif Ul Islam, Stuart Harrison, Gregory Epiphaniou, Nikolaos Matragkas, Theodoros N. Arvanitis, Carsten Maple

**Affiliations:** 1 Warwick Manufacturing Group, University of Warwick, Coventry, CV4 7AL UK; 2 ETHOS Digital Health Ltd, Exeter, EX4 3SR UK; 3 CEA List, Université Paris-Saclay, 2 Bd Thomas Gobert, 91120 Palaiseau, France; 4 Department of Electronic, Electrical and Systems Engineering, School of Engineering, University of Birmingham, Edgbaston, Birmingham, B15 2TT UK

**Keywords:** Digital health, Risk assessment, Healthcare data categories, Healthcare data modalities, Cybersecurity, Patient safety, Biomarkers, Health care economics, Health policy, Medical ethics, Public health, Quality of life, Information technology, Scientific data, Statistics

## Abstract

The increasing digitization of healthcare data systems presents substantial opportunities for enhancing patient care and operational efficiency, while simultaneously introducing critical vulnerabilities such as unauthorized access, inconsistent data formats, and privacy breaches. To systematically address these risks, this study employs Failure Modes and Effects Analysis (FMEA) to identify, evaluate, and prioritize potential hazards within digital healthcare systems. It is among the first to apply the FMEA approach in a comprehensive manner to assess risks across diverse healthcare data categories and modalities, offering a novel perspective on the vulnerabilities inherent in digital health systems. Through a structured methodology, this research investigates risks across three key healthcare data categories, such as clinical, operational, and patient-reported, as well as across five major data modalities including text, image, tabular, audio, and video. Each identified failure mode was assessed through expert consultation and comprehensive literature review, considering its severity, occurrence, and detectability, and subsequently assigned a Risk Priority Number for quantitative prioritization. Key findings highlighted significant risks, including unauthorized access, data corruption, transmission errors, and privacy breaches, that threaten patient safety and system reliability. This study provides actionable recommendations to strengthen data integrity, security, and interoperability, supporting the safe adoption of AI, blockchain, and other emerging technologies in developing secure and resilient digital healthcare systems.

## Introduction

The digital transformation of healthcare has resulted in a substantial increase in the volume and diversity of data types, including Electronic Health Records (EHRs), medical imaging, genomic sequences, and patient-generated data^1,2^. Worldwide, adoption is now widespread, such as 96% of acute-care hospitals in the United States and more than 80% of OECD countries use certified EHR platforms, with the forthcoming European Health Data Space set to enable cross-border exchange of clinical data^3^.

EHR is a digital collection of patient health information, such as medical history, diagnoses, medications, treatment plans, immunization dates, allergies, radiology images, and laboratory test results, that is securely shared among authorized providers to support patient care^4^. This wealth of information holds the potential to enhance clinical decision-making, personalize treatments, and improve health outcomes. Big Data Analytics (BDA) in healthcare enables the extraction of meaningful insights from vast datasets, facilitating early disease detection, resource optimization, and evidence-based policymaking^1^.

**Fig. 1:**
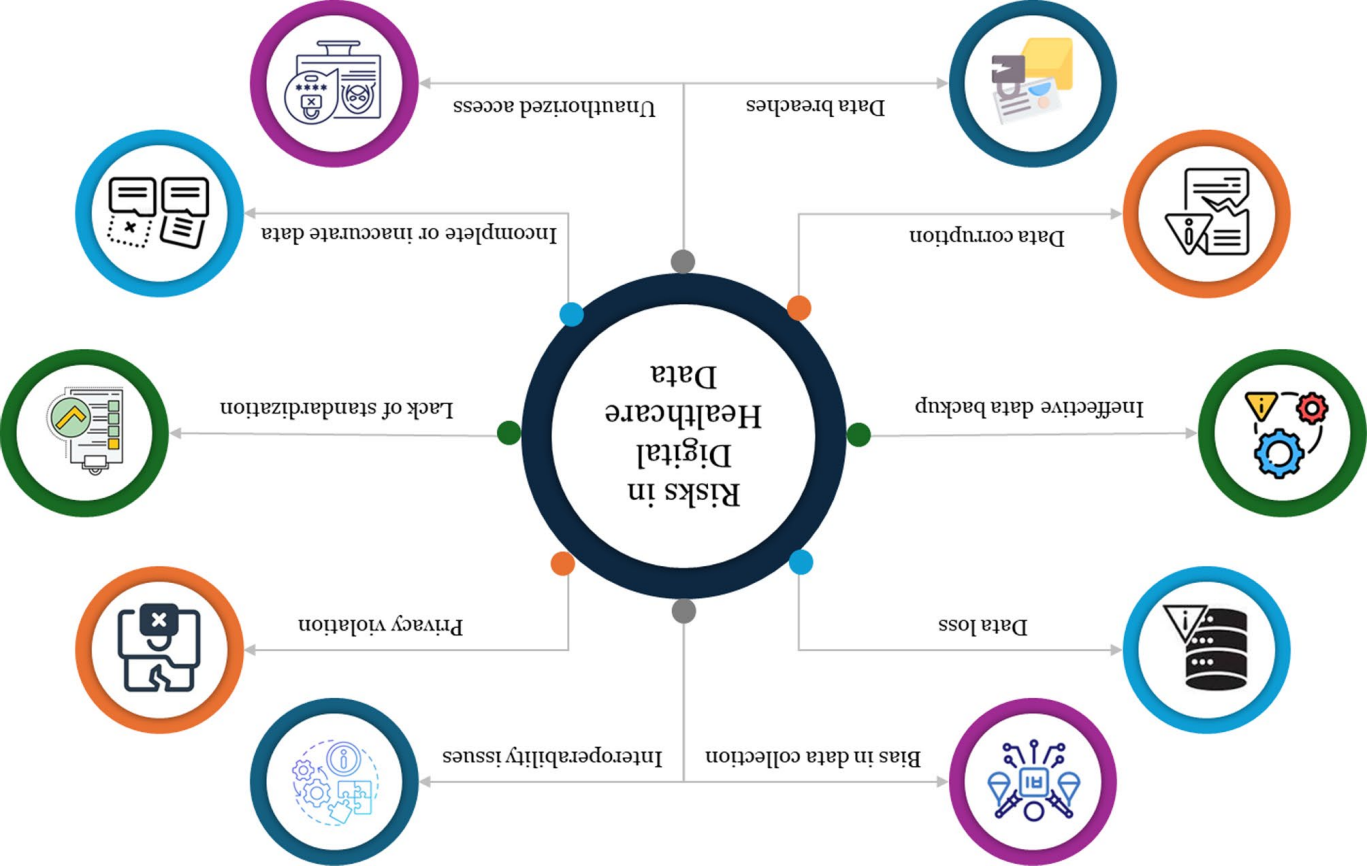
Hazards in digital healthcare data.

Despite these advancements, managing healthcare data presents significant challenges, highlighted in Fig. 1. Data quality issues, such as incompleteness, inconsistency, and inaccuracies, can compromise patient safety and delay effective care delivery. Moreover, concerns about data privacy and security are paramount, as healthcare data breaches can lead to severe consequences for individuals and institutions. The 2017 WannaCry ransomware attack, which disrupted more than 40 hospitals in England’s National Health Service and forced staff to revert to paper records, exemplifies the scale of harm that cyber-vulnerabilities can inflict^5^. The heterogeneity of data sources and formats further complicates data integration and interoperability, delaying seamless information exchange across healthcare systems^6^. Table 1 comparing recent works, highlighting each method’s functionality and novelty, and identifying gaps we need to address in this study.

To address these challenges, systematic risk assessment methodologies are essential^17^. FMEA is a proactive approach that identifies potential failure points within a system, assesses their impact, and prioritizes them based on severity, occurrence, and detectability. Originally developed in the manufacturing sector, FMEA has been adapted for healthcare to enhance patient safety and improve process reliability^13^. Its application in healthcare settings allows for the anticipation and mitigation of risks before adverse events occur^18^.

While the adoption of digital technologies in healthcare has improved data accessibility and patient engagement, it has also introduced new vulnerabilities^1^. The complexity of healthcare data systems, coupled with the critical nature of the information they handle, necessitates a comprehensive approach to risk management. This study addresses the gap in systematic risk assessment of healthcare data management by employing FMEA to identify, evaluate, and prioritize potential data-related hazards. By integrating insights from academic literature, technical standards, industry practices, and expert consultations, the study aims to provide a structured framework for enhancing data safety, quality, and operational efficiency in digital healthcare systems. Accordingly, the core objectives of this study are to:

- Develop an FMEA-based framework specifically tailored to the heterogeneous data flows of EHR-centered healthcare systems;
- Apply the framework in real-world applications to identify and prioritize critical failure modes based on severity, occurrence, and detectability; and.
- Propose evidence-based mitigation strategies that enhance data quality, privacy, security, and interoperability, while remaining feasible within routine clinical workflows.

The remainder of this paper is organized as follows: Section 2 details the methodology, including scope definition, literature review, expert consultation, FMEA-based risk assessment, risk prioritization, and practical implications. Section 3 presents the results of the FMEA, highlighting identified failure modes and their corresponding risk priority numbers. Section 4 discusses the findings, offering insights into key vulnerabilities and recommendations for mitigation and directions for future research.

**Table 1:** Comparative overview of recent studies applying risk assessment and predictive analytics methods in digital healthcare.

Study	Methods	Significance	Limitations
Kiourtis et al.^7^.	Gaia-X/IDS-inspired cross-sector data space linking environmental sensors with EHR data to enable federated risk queries.	First blueprint combining environmental and health domains for predictive analytics.	Proof-of-concept only; single pilot; no large-scale validation.
Montella et al.^8^.	Comparative review of Enterprise Risk Management, FMEA, RCA, Swiss-Cheese and Bowtie into a system-wide patient-safety framework.	Holistic roadmap for selecting and sequencing risk-management tools.	Purely theoretical; lacks empirical demonstration.
En-Naaoui et al.^9^.	Fuzzy-FMEA with a new Control factor led to fuzzy inference for RPN, ANN for tolerance classes, SVM for action priority; sterilization-unit pilot.	Shows AI can overcome classical-FMEA limits, hitting ≥ 98% classification accuracy.	Single department; no long-term or multi-site validation.
Tangestani et al.^10^.	FMEA with Decision-Matrix Risk Assessment to rank 19 bio-aerosol failure modes in four hospitals; reliability tests (α = 0.619, ICC = 0.913).	First hospital-level protocol for indoor-air-pollutant risk using FMEA.	Four Iranian hospitals; no follow-up outcomes; moderate internal reliability
Li Hu et al.^11^.	FMEA of dental-clinic needlestick hazards; pre/post intervention RPN comparison over 12 months.	RPNs cut ≥ 75%; injury rate fell from 21.6–5.4%.	Single clinic; 37 staff; no multi-centre replication.
Mavrogiorgos et al.^12^.	Systematic review mapping bias sources and more than 180 mitigation techniques across the ML lifecycle.	First end-to-end taxonomy linking bias origins to concrete fixes.	Descriptive; engineer-bias under-represented; no benchmarking.
Vecchia et al.^13^.	PRISMA-ScR scoping review of around 163 studies on FMEA in infection-control contexts.	Consolidates evidence, exposes gap (< 8%) in infection-prevention FMEA work.	Language and peer-review filters; heterogeneity excluded meta-analysis.
Mavrogiorgou A. et al.^14^.	Scoping review and decision tree categorization for more than 100 ML healthcare risk prediction algorithms.	Ready reference linking algorithms to clinical risk tasks.	No head-to-head benchmarking.
Sharbati et al.^15^.	FMEA of medication-management workflow with Pareto analysis; pre/post RPN reduction.	FMEA-Pareto combo cut top RPNs by 60%, template for low-resource settings.	Single inpatient unit; short follow-up; no patient-outcome data.
Reščič et al.^16^.	SmartCHANGE protocol: federated, explainable DL on 15 datasets (≈ 200 k youths) in an HL7-FHIR data space for NCD risk prediction.	First EU-wide privacy-preserving framework for youth NCD risk and behavior change.	Methodology paper; no results; success depends on multi-year pilots.

## Methodology

**Fig. 2:**
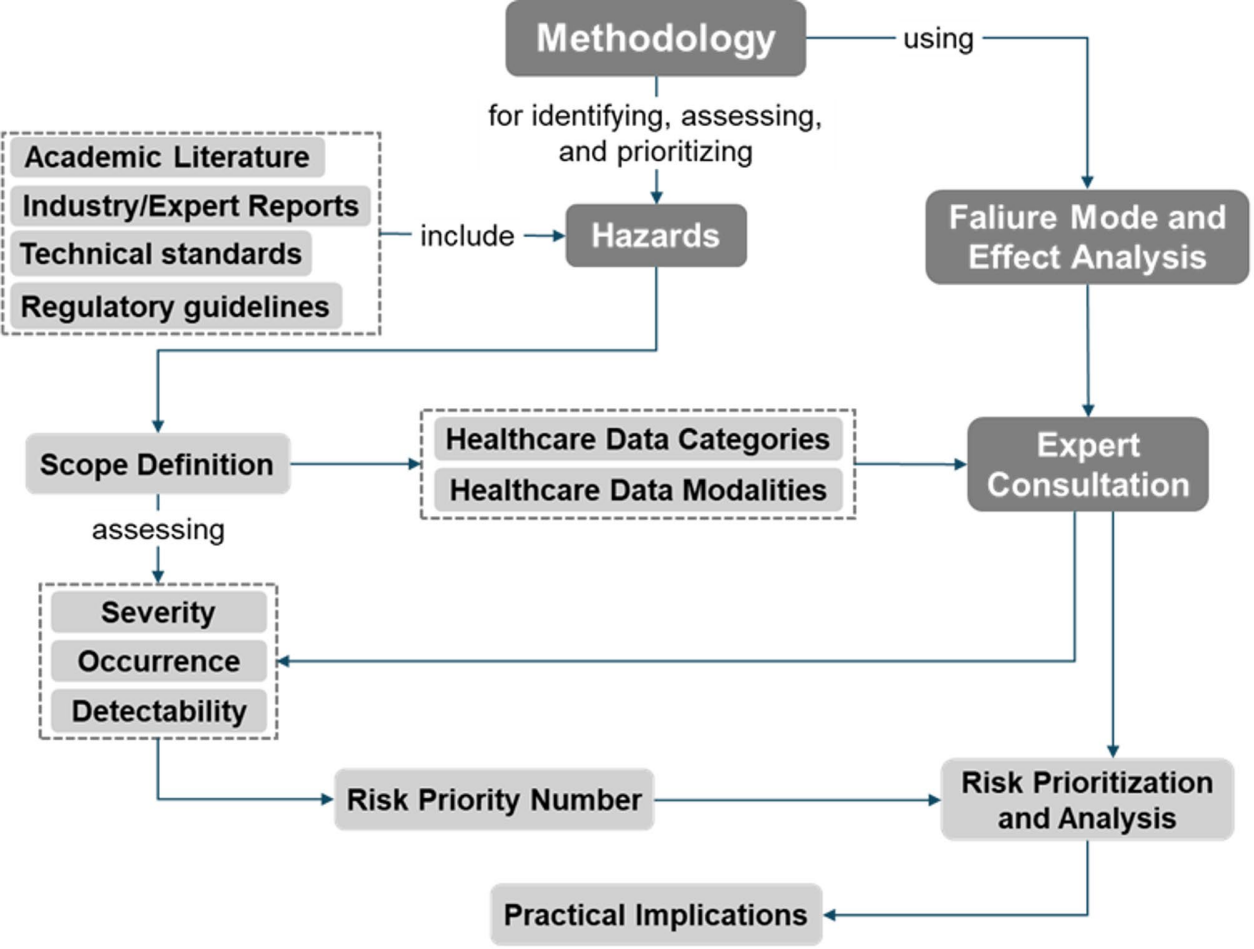
Methodology for hazard identification and risk evaluation across healthcare data categories and modalities through FMEA.

This study employed a structured, expert-driven approach to identify, assess, and prioritize data-related hazards in digital healthcare systems using the FMEA framework. The methodology integrates evidence from academic literature, technical standards, industry best practices, and expert consultation to ensure both scientific consistency and real-world relevance. A visual representation of the methodological framework is depicted in Fig. 2. The overall sequence of activities was guided by a modified Delphi consensus process, while the literature search followed PRISMA-ScR recommendations, ensuring that each step was evidence-based and refined through successive expert feedback^19^.

### Scope definition

**Fig. 3:**
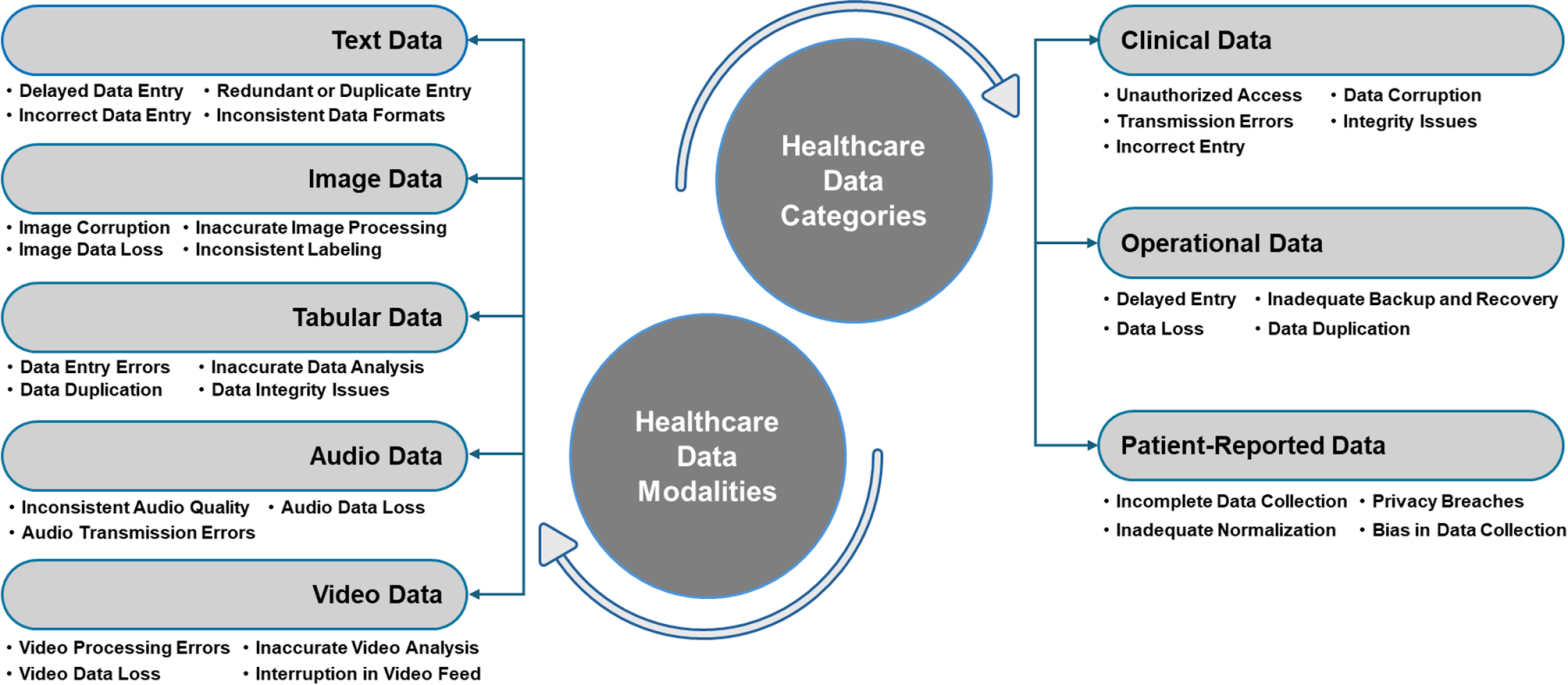
Risks across healthcare data categories and modalities.

A clearly defined and comprehensive scope was established at the outset of the study. The analysis was structured along two primary axes, outlined in Fig. 3, such as:

- Healthcare data categories: The analysis focused on three primary categories, such as clinical data, operational data, and patient-reported data, to capture the diverse roles of data in healthcare.
- Healthcare data modalities: Five widely used data formats, such as text, image, tabular, audio, and video, were selected due to their relevance across digital health systems.

These domains were selected due to their importance to patient care delivery, and the broader digital health ecosystem. This comprehensive scope ensured that FMEA addressed both the content and structure of healthcare information.

### Literature review and expert consultation

A systematic review of authoritative and reliable sources was conducted to identify failure modes and establish a foundation for risk evaluation. This review included:

- Peer-reviewed literature: Research studies on health IT risks, data quality, AI in healthcare, and information governance were reviewed to identify frequent hazards and evaluate their implications. Table 1 highlights a few studies.
- Industry reports: Publications from healthcare technology providers and cybersecurity organizations offered practical insights into evolving threats and operational weaknesses.
- Technical standards and regulatory guidelines: Frameworks such as HL7, FHIR, ISO, HIPAA, and GDPR were referenced to ensure regulatory alignment and consistent interpretation of risk factors. Relevant EHR standards, including HL7 FHIR, openEHR, and ISO/EN 13,606, were also reviewed to ensure that the risk assessment framework aligns with established interoperability and data exchange requirements.
- Data collection and preprocessing: No primary patient-level dataset was analysed; instead, we compiled a review of more than 100 peer-reviewed studies, 15 technical standards and 9 incident-report datasets that describe data-related failures in digital health. Duplicate records were removed with rule-based matching and Jaccard/Cosine similarity thresholds following Kiourtis et al.’s Data-Reliability micro-service pattern^7^. Missing or inconsistent metadata were imputed or harmonized according to the bias-mitigation taxonomy of Mavrogiorgos et al.^12^, ensuring that downstream FMEA scoring was not skewed by selection or reporting bias.

This triangulated review enabled the foundation of a robust knowledge base for evaluating the severity, likelihood, and detectability of healthcare data failures. In addition to validating and contextualizing the findings derived from the literature, consultations with domain experts were conducted across various fields, including health information management, healthcare IT and cybersecurity, and data science and analytics. These expert sessions, held through workshops and review panels to validate the identified list of failure modes across different categories and modalities, assign risk scores (Severity, Occurrence, and Detectability) based on operational experience, and determine realistic thresholds for the Risk Priority Number (RPN). These discussions were essential in prioritizing risks within the context of operational healthcare environments.

### FMEA-based risk assessment

The FMEA framework served as the core risk assessment methodology, enabling a structured evaluation of failure points within digital healthcare systems. Each identified failure mode was assessed across three factors:

- Severity (S): The potential impact of failure on patient outcomes, data integrity, and system performance.
- Occurrence (O): The estimated frequency of the failure under normal operating conditions.
- Detectability (D): The likelihood of detecting the failure before it results in adverse consequences.

Each factor was scored on a scale of 1 to 10, according to the interpretation thresholds shown in Table 2. Higher scores indicated more severe consequences, more frequent occurrences, or lower chances of detection. The RPN for each failure mode was calculated as, RPN = S × O × D. This quantitative value enabled objective comparison across risks and provided a structured basis for prioritization. However, all RPN calculations and visualisations were performed in excel sheets because its transparency and ubiquity facilitate audit by non-technical stakeholders; no custom code or specialised ML libraries were required, reflecting the expert-driven, rather than algorithmic, nature of the present FMEA.

### Risk prioritization and analysis

After evaluating the RPNs, risks were ranked and classified into priority levels, such as moderate, high, or critical, based on both numerical values and expert assessment. This step translated the quantitative findings into actionable insights by considering: (1) the proximity of each risk to patient care, data security, and regulatory concerns; (2) historical relevance or known cases of similar failures within the healthcare domain; and (3) resource and operational considerations required for addressing each risk effectively.

This prioritization framework supported the development of targeted mitigation strategies that balanced impact, feasibility, and resource allocation. The final risk rankings were reviewed and validated by domain experts to ensure alignment with practical constraints and system-level priorities.

### Practical implications and future scoping

The final phase of the methodology focused on transforming the findings into actionable insights and long-term strategies aimed at supporting resilience in healthcare data management. Recommendations were formulated for key healthcare stakeholders, including EHR developers, clinical administrators, and policymakers. These recommendations emphasized strengthening the reliability and traceability of EHR systems, securing patient-generated data streams, improving the resilience of telemedicine systems, and standardizing data formats to enhance interoperability across various digital systems.

In addition to these practical implications, forward-planning efforts were carried out in consultation with domain experts to support innovation and strategic growth. These initiatives include incorporating AI-driven data validation within clinical workflows, leveraging advanced cybersecurity technologies such as blockchain to develop more secure data environments, and enabling personalized medicine through the use of high-quality, standardized, and bias-mitigated data. Together, these outcomes ensure that the methodology not only assesses risk but also contributes to informed decision-making, promotes innovation, and strengthens the long-term resilience of digital health systems.

**Table 2:** FMEA-based risk assessment.

Score	Severity Interpretation	Occurrence Interpretation	Detectability Interpretation
1–3	Low	Rare	Easily detectable
4	Low to Medium	Rare to Medium	Easy to Medium
5–6	Medium	Medium	Medium
7	Medium to High	Medium to High	Medium to Hard
8–9	High	High	Hard to detect/Low detectability
10	Extremely Severe	Frequent	Almost undetectable

## Results

This section details the outcomes from applying FMEA to identify, evaluate, and prioritize hazards associated with healthcare data management systems. The analysis addresses specific risks across defined healthcare data categories and modalities, highlighted in Fig. 3, emphasizing their criticality to patient safety, operational continuity, and clinical outcomes. Each identified failure mode was quantitatively assessed based on Severity, Occurrence, and Detectability, resulting in a Risk Priority Number, helps prioritize risks based on their potential impact, likelihood, and difficulty of detection. Results are summarized in detailed Tables 3 and 4 to facilitate practical insights and informed decision-making.

### Risk evaluation across key healthcare data categories

Healthcare data are broadly classified into distinct categories based on their primary usage and impact on healthcare delivery. For this analysis, three essential healthcare data categories were selected: Clinical Data, Operational Data, and Patient-Reported Data. These categories collectively encompass the breadth of healthcare operations, directly influence patient care outcomes, and represent core areas for potential data management risks. Each data category demonstrated distinct risks with varying RPN values, summarized comprehensively in Table 3.

#### Clinical data

Clinical data is central to healthcare, forming the foundation for accurate diagnoses, effective treatment, and evidence-based practice. It encompasses patient medical records, diagnostics, lab results, and treatment histories, essential for clinical decision-making and patient safety. However, its sensitive nature exposes it to several risks:

- Unauthorized access: Unauthorized access emerged as the most critical risk, driven by inadequate password management, phishing attacks, and weak access controls. Such breaches can lead to identity theft, compromised patient confidentiality, and erroneous clinical decisions, all of which pose significant threats to patient safety and institutional reputation^20–22^. The severity of unauthorized access is rated high (~ 9) due to significant potential harm to patient confidentiality and accuracy of clinical decisions. The likelihood of occurrence is also high (~ 8), exacerbated by frequent cybersecurity threats and phishing incidents. Detectability is considered medium (~ 6), as standard security tools can identify typical breaches, although more sophisticated attacks may initially evade detection.
- Data corruption: Data corruption, often caused by malware, improper data handling, or system malfunctions, significantly impacts clinical outcomes due to diagnostic inaccuracies and delayed treatments^23,24^. The severity of data corruption is rated high (~ 8), given its direct impact on clinical decision accuracy and patient treatment outcomes, reported by Nemec et al.^23^. The occurrence is moderate to high (~ 6), commonly resulting from software vulnerabilities, hardware failures, or cybersecurity threats. Detectability is challenging (~ 8), as minor corruptions often remain overlooked without sophisticated and continuous monitoring mechanisms. Mejía-Granda et al.^24^ highlight vulnerabilities such as poor credential management and software vulnerabilities, emphasizing this risk.
- Incorrect data entry: Incorrect data entry is prevalent in healthcare, particularly in systems reliant on manual inputs. Such errors significantly threaten patient safety by causing misdiagnoses or inappropriate treatments. The severity of incorrect data entry is rated high (~ 8), supported by reports from the ECRI Institute indicating that human-computer interface issues constitute 65% of health IT-related incidents^25^. Occurrence is moderate to high (~ 6), especially prevalent in high-pressure environments where manual data entry is common. Detectability is medium (~ 5), generally facilitated by validation checks or cross-verification procedures, though minor errors may occasionally remain undetected^26^.
- Data transmission errors: Errors occurring during data transmission can lead to significant delays or inaccuracies in clinical communication, impacting patient care directly. Anne Trafton^27^ reports that such errors have led to breaches affecting nearly 100 million individuals, indicating high severity (~ 8). Occurrence is medium to high (~ 7), dependent upon network complexity and robustness of IT infrastructure. Detectability is typically medium (~ 4), as robust monitoring tools can flag common transmission errors effectively, though certain errors can initially bypass detection^28^.
- Data integrity issues: Failures in maintaining data integrity represent critical hazards with potentially severe clinical consequences, arising from human errors, system anomalies, or undetected cyber manipulations. Data integrity failures, including unauthorized modification or unnoticed corruption, undermine the reliability of clinical decisions^29^. Severity is therefore high (~ 9), reflecting the significant potential harm to patient safety and clinical reliability. Occurrence is also high (~ 8), owing to frequent vulnerabilities and cyber threats prevalent in healthcare IT infrastructures. Detectability is moderate to high (~ 3), as routine validation protocols, real-time system checks, and advanced cybersecurity measures generally aid early detection.

#### Operational data

Operational data encompasses administrative and logistical information essential for managing healthcare systems efficiently. This data category includes staffing schedules, resource allocation details, compliance management, and supply chain data, each essential for the effective delivery of healthcare services.

- Delayed data entry: Delayed data entry is a significant risk, primarily due to high workloads, inadequate training, or reliance on manual processing. The report published by ECRI institute highlighted that such delays disrupt patient care continuity and reduce operational efficiency, potentially leading to increased costs and compromised patient outcomes^25^. Severity is high (~ 8), reflecting its direct impact on healthcare service effectiveness. Occurrence is moderate to high (~ 6), particularly common during peak workloads or manual administrative processes, whereas, detectability is low (~ 8), often challenging without automated auditing or regular monitoring practices^26^.
- Inadequate backup and recovery: Insufficient redundancy, outdated systems, or inadequate assessment of backup protocols contribute significantly to this risk. Failures in backup and recovery processes lead to severe data loss, operational downtime, and regulatory non-compliance^30^. Severity is rated high (~ 10), given the profound implications for institutional operations and compliance. Occurrence is moderate (~ 5), dependent on organizational adherence to recommended practices. Detectability is also moderate (~ 5), typically identified during routine testing, although certain failures become apparent only during critical recovery processes.
- Data loss: Data loss, resulting from system failures, cyberattacks, or physical damage to storage devices, severely affects healthcare operational continuity and incurs substantial legal and regulatory consequences^31,32^. Argaw et al.^31^ emphasize the impact of cyberattacks, such as the WannaCry incident, which underscored the vulnerabilities of healthcare systems. Severity of such hazard is therefore high (~ 9), considering the irreversible nature and critical impact on operations. Occurrence is moderate to high (~ 7), frequently associated with cyber threats and inadequate protective measures. However, detectability is moderate to high (~ 3), generally becoming apparent immediately in critical events, but low-visibility or gradual data leaks may remain initially overlooked.
- Data duplication: Data duplication results primarily from poor synchronization, manual entry errors, or inadequate system integration. Although typically less severe than data corruption or loss, Acceldata’s report on data quality highlighted that duplication leads to confusion among healthcare providers, increased storage costs, and potential clinical inaccuracies^33^. Severity is moderate to high (~ 7), considering the indirect impact on patient care and operational efficiency. Occurrence is also high (~ 7), especially in complex or interoperable healthcare environments. Detectability is relatively high (~ 3), typically easy to identify through data management or deduplication tools.

**Table 3:** FMEA for healthcare data categories.

	Hazard Mode	Potential Causes	Consequences	RPN	Priority Level
Clinical Data	Unauthorized Access	Weak security, phishing	Data breaches, identity theft	432	High
	Data Corruption	Malware, system errors	Misdiagnoses, delayed treatments	384	High
	Incorrect Data Entry	Human errors, interface issues	Misdiagnoses, incorrect treatments	240	Moderate
	Data Transmission Errors	Network/system incompatibility	Delays or inaccuracies in treatment	224	Moderate
	Data Integrity Issues	Unauthorized modifications, cyber threats	Clinical inaccuracies, compromised trust	216	Moderate
Operational Data	Delayed Data Entry	High workload, manual processes	Operational disruptions, delayed care	384	High
	Inadequate Backup and Recovery	Lack of redundancy, inadequate testing	Data loss, non-compliance	250	High
	Data Loss	Cyberattacks, system failures	Operational disruptions, compliance issues	189	Moderate
	Data Duplication	Poor synchronization, manual entry errors	Operational inefficiency, increased costs	147	Moderate
Patient-Reported Data	Privacy Breaches	Insider threats, weak encryption	Identity theft, loss of patient trust	640	Critical
	Incomplete Data Collection	Poor protocols, patient non-compliance	Misdiagnoses, inappropriate treatments	432	High
	Bias in Data Collection	Selective reporting, demographic biases	Inequitable healthcare outcomes	336	High
	Inadequate Data Normalization	Inconsistent formats, lack of standardization	Analytical errors, clinical misinterpretations	320	Moderate

*S: *1 (least severe) to 10 (most severe), *O: *1 (rare) to 10 (frequent), *D: *1 (easily detectable) to 10 (hard to detect).

#### Patient-reported data

Patient-reported data encompasses health information provided directly by patients, including symptoms, treatment adherence, and quality-of-life measures. Such data is critical for personalized care and patient engagement but presents unique risks due to its subjective nature, sensitivity, and variable quality.

- Privacy Breaches: Privacy breaches represent the most critical risk, often resulting from insider threats, inadequate encryption, or weak monitoring systems. Breaches can cause identity theft, loss of patient trust, significant legal repercussions, and damage to institutional reputation^34–36^. The severity of such breaches is very high (~ 10), reflecting profound consequences on patient trust and institutional integrity. Occurrence is also high (~ 8), frequent due to ongoing vulnerabilities and insider threats, whereas the detectability is often low (~ 8), particularly challenging in anonymized or aggregated datasets.
- Incomplete Data Collection: Incomplete data collection arises from poorly designed protocols, patient non-compliance, or technical limitations. Missing patient-reported information severely impacts clinical accuracy, leading to potential misdiagnoses or inappropriate treatments and reduced patient satisfaction^25,26^. A notable case involved incorrect dosing due to incomplete data in computerized provider order entry systems^25^. Severity of such an incident is high (~ 9), due to direct clinical implications, whereas the occurrence is moderate (~ 6), frequently occurring with manual or poorly structured data collection systems. Detectability is relatively low (~ 8), requiring specialized completeness audits and validation tools^37,38^.
- Bias in Data Collection: Data collection bias, resulting from selective reporting, underrepresentation, or inadequate demographic sampling, leads to inequitable healthcare outcomes and distorted analytical insights^4,39^. Severity is high (~ 8), as it directly affects equity in healthcare delivery and decision-making. Occurrence is moderate (~ 6), often associated with patient demographics and collection methodologies, whereas detectability is moderate to low (~ 7), necessitating complex statistical analyses and vigilance in data collection practices.
- Inadequate Data Normalization: Lack of standardized data normalization, caused by inconsistent formats or varying collection standards, contributes to misinterpretation and clinical errors^38^. The 4medica report highlighted its high severity (~ 8), reflecting its critical impact on data usability and analytical accuracy^38^. Occurrence is moderate (~ 5), common in diverse healthcare settings with multiple systems or protocols, whereas detectability is often low (~ 8), as detection requires careful validation and standardized analytical tools.

### Risk evaluation across healthcare data modalities

Healthcare data modalities, including text, image, tabular, audio, and video data, represent varied formats used to capture, store, and process critical health-related information. Each modality has distinct technical properties, clinical relevance, and vulnerabilities, influencing its susceptibility to specific types of hazards. Due to their unique characteristics and applications in healthcare, these modalities require tailored risk assessment and management strategies. This section extends the application of the FMEA framework by systematically evaluating risks specific to each data modality. The identified failure modes, their underlying causes, potential consequences, and calculated RPNs for each modality are comprehensively summarized in Table 4.

#### Text data

Text data, including clinical notes, prescriptions, patient histories, and discharge summaries, forms the foundational element of healthcare documentation, clinical decision-making, and communication. However, its critical role makes it particularly vulnerable to several risks, including incorrect and delayed data entry (discussed above), as well as redundant or duplicate entries and inconsistent data formats.

- Redundant or Duplicate Entry: Redundant or duplicate entries result from manual repetition, poor system synchronization, or integration errors. Duplication creates clinical confusion, contributes to medical errors, and increases healthcare operational costs. Studies, such as those by Verato and AHIMA, report that 20% of medical records in multi-facility systems are duplicates, costing up to $40 million for providers annually^40,41^. Severity is moderate to high (~ 7), as duplication indirectly affects patient care and clinical efficiency. Occurrence is also moderate to high (~ 7), frequently observed in healthcare settings lacking robust integration or deduplication mechanisms. Detectability is often high (~ 3), usually straightforward with automated validation or deduplication software^26^.
- **I**nconsistent Data Formats: Lack of standardized text data formats, due to inconsistent system implementations or absence of standardized protocols (e.g., HL7, FHIR variations), leads to interoperability challenges, clinical misinterpretation, and information loss^42,43^. Severity is moderate to high (~ 7), impacting effective data utilization and clinical communication. Occurrence is also high (~ 7), common in multi-system environments lacking strict standard adherence. Detectability is high (~ 3), as validation and interoperability checks can promptly identify inconsistent formatting.

#### Image data

Image data, encompassing radiographs, MRIs, CT scans, and ultrasounds, is vital for medical diagnostics, treatment planning, and patient care monitoring. Despite its diagnostic importance, it faces significant risks:

- Inaccurate image processing: Algorithmic errors, biased training datasets, or faulty analysis models often cause inaccurate image processing, leading to incorrect diagnostic outcomes, mismanagement of conditions, and potential patient harm^42^. The severity of inaccurate processing is very high (~ 10), reflecting its direct and potentially severe impact on clinical decision-making, whereas, the occurrence of this risk is moderate (~ 5), increasing with broader adoption of automated image analysis technologies. The detectability is often low (~ 8), requiring rigorous validation protocols to reliably detect errors.
- Image corruption: Image corruption occurs primarily due to transmission errors, network failures, or faulty storage mechanisms. Corrupted images result in incomplete or unreadable diagnostic information, significantly affecting clinical accuracy and patient care quality^23^. It has high severity level (~ 8), given the direct implications for diagnosis reliability, with medium to high likelihood (~ 7), frequent due to varying network reliability and storage practices. Detectability is often medium (~ 5), typically detectable during routine image review or quality assurance procedures.
- Image data loss: Loss of critical medical imagery, caused by inadequate backup practices, storage failures, or cyber incidents, severely disrupts clinical workflows and diagnostic continuity^31^. The severity of such data loss is high (~ 9), as lost images can significantly compromise patient care. The occurrence is medium to high (~ 7), common in healthcare environments lacking robust data redundancy systems. Detectability of such image data loss is often high (~ 3), typically evident immediately following major data loss incidents but challenging for subtle or incremental data losses.
- Inconsistent labelling: Inconsistent image labelling, arising from manual errors or inadequate labelling standards, leads to misclassification or confusion in diagnostic processes^42,43^. Severity is moderate (~ 7), due to its indirect yet significant impact on diagnosis accuracy and patient outcomes. However, the occurrence of inconsistent labelling is usually medium (~ 6), dependent on institutional adherence to labelling guidelines. Detectability is also medium (~ 5), identifiable through standardized audits or image review processes.

#### Tabular data

Tabular data, including laboratory results, patient vitals, and resource management data, is essential for clinical analytics, decision support, and healthcare operations management. Risks affecting this modality include data duplication, integrity, and entry errors (discussed above), as well as inaccurate data analysis.

- Inaccurate data analysis: Algorithmic biases, incorrect statistical modelling, or flawed analytical approaches contribute significantly to inaccurate tabular data analysis, causing misguided clinical and operational decisions^44^. The severity of inaccurate analysis is high (~ 8), directly influencing the effectiveness and accuracy of decision-making processes. Occurrence is often medium (~ 6), increasing with reliance on automated or algorithmic data analytics, with low detectability (~ 8), requiring careful statistical validation or cross-validation to identify inaccuracies effectively.

**Table 4:** FMEA for healthcare data modalities.

	Hazard Mode	Potential Causes	Consequences	RPN	Priority Level
Text Data	Delayed Data Entry	Manual processing, inefficient systems	Delayed treatment, outdated information	384	High
	Incorrect Data Entry	Manual errors, software bugs	Misdiagnosis, inappropriate treatments	240	Moderate
	Redundant or Duplicate Entry	Manual repetition, poor synchronization	Clinical confusion, increased costs	147	Moderate
	Inconsistent Data Formats	Lack of standardized protocols	Data misinterpretation, loss of information	147	Moderate
Image Data	Inaccurate Image Processing	Algorithm errors, biased datasets	Incorrect diagnostics, treatment errors	400	High
	Image Corruption	Transmission errors, storage issues	Unreadable diagnostic images, clinical errors	280	Moderate
	Inconsistent Labeling	Manual errors, inadequate standards	Misclassification, diagnostic confusion	210	Moderate
	Image Data Loss	Inadequate backups, storage failures	Lost critical diagnostic information	189	Moderate
Tabular Data	Inaccurate Data Analysis	Algorithm biases, incorrect modeling	Misguided decisions, clinical errors	384	High
	Data Entry Errors	Manual inputs, poor validation	Incorrect patient records, clinical errors	240	Moderate
	Data Integrity Issues	Unauthorized modifications, cyberattacks	Clinical inaccuracies, compromised decisions	216	Moderate
	Data Duplication	Poor integration, manual processes	Increased operational costs, confusion	147	Moderate
Audio Data	Inconsistent Audio Quality	Recording issues, compression artifacts	Diagnostic errors, clinical misunderstandings	245	Moderate
	Audio Transmission Errors	Network interruptions, technical failures	Loss of critical clinical information	192	Moderate
	Audio Data Loss	System crashes, inadequate storage	Incomplete clinical documentation	189	Moderate
Video Data	Video Processing Errors	Faulty AI models, biased training	Misinterpretation, clinical errors	392	High
	Inaccurate Video Analysis	Model overfitting, inadequate training	Misclassification, inappropriate decisions	320	Moderate
	Video Data Loss	Storage/network failures	Disruption of clinical workflow	270	Moderate
	Interruption in Video Feed	Network instability, equipment malfunctions	Interrupted patient monitoring, delayed interventions	140	Moderate

*S*: 1 (least severe) to 10 (most severe), *O*: 1 (rare) to 10 (frequent), *D*: 1 (easily detectable) to 10 (hard to detect).

#### Audio data

Audio data, such as recorded consultations, heart and lung sounds, and patient assessments, is essential for detailed patient evaluation and clinical diagnostics. However, audio data management faces significant risks:

- Inconsistent audio quality: Poor audio quality, arising from recording equipment issues, background interference, or compression artifacts, significantly affects clinical interpretation accuracy^28^. Severity of such issues is moderate to high (~ 7), potentially leading to diagnostic errors or misunderstandings. Occurrence is medium (~ 5), often related to equipment standards or recording practices. Detectability is moderate (~ 7), typically identified through routine audio quality assessments or reviews.
- Audio transmission errors: Errors in audio transmission, caused by network interruptions, technical failures, or inadequate bandwidth, result in incomplete or unclear clinical information delivery^27,28^. Anne Trafton^27^ reports that such errors have led to healthcare data breaches affecting nearly 100 million people, underscoring their high severity (~ 8). Occurrence is medium (~ 6), frequently influenced by network infrastructure reliability. Detectability is moderate (~ 4), identifiable through monitoring systems or real-time network checks.
- Audio data loss: Loss of critical audio data due to storage failures, system crashes, or inadequate backups severely affects clinical documentation completeness and accuracy^27^. Severity of such loss is high (~ 9), due to its direct impact on clinical accuracy and patient records integrity. Occurrence is medium to high (~ 7), depending on storage robustness, whereas detectability is moderate (~ 3), typically noticeable following significant data loss events.

#### Video data

Video data significantly enhances clinical care through telemedicine consultations, surgical recordings, patient monitoring, and procedural documentation. It provides comprehensive visual information essential for accurate clinical assessments and decision-making but presents several vulnerabilities:

- Video processing errors: Video processing errors, often resulting from misconfigured AI models, biased algorithm training, or inadequate software validation, severely affect clinical evaluations by causing misinterpretations or incorrect diagnostic outcomes^27^. The severity of such errors is rated high (~ 8), reflecting their potential to lead directly to incorrect patient assessments or treatments. Occurrence is moderate to high (~ 7), particularly prevalent with increased reliance on automated video-processing algorithms. Detectability is moderate to low (~ 7), since detection typically requires rigorous validation or extensive manual verification processes.
- Inaccurate video analysis: Inaccurate analysis of video data frequently arises from model overfitting, inadequate training datasets, or poorly calibrated analytical tools, causing misclassification or misinterpretation of clinical scenarios captured on video. These inaccuracies can lead to flawed clinical decisions, inappropriate interventions, or patient safety risks^27^. Severity is moderate to high (~ 8), due to the direct implications for patient care outcomes. Occurrence is moderate (~ 5), dependent on the maturity of the video analysis technology used. Detectability is moderate to low (~ 8), as identification typically involves complex clinical validation or repeated manual reviews.
- Video data loss: Loss of critical clinical video data due to storage or network failures, inadequate backup solutions, or cyber incidents significantly disrupts clinical workflow, documentation, and patient monitoring processes^31,32^. The severity of video data loss is high (~ 9), considering its potential to severely disrupt clinical evaluations and documentation continuity. Occurrence is moderate (~ 6), especially in institutions with suboptimal data redundancy measures or outdated storage infrastructure. Detectability is also moderate (~ 5), usually apparent after critical data-loss incidents occur, though non-apparent incremental losses may initially remain overlooked.
- Interruption in video feed: Interruptions in video feeds, often caused by network instability, equipment malfunctions, or bandwidth limitations, negatively impact real-time clinical monitoring, telemedicine sessions, and remote patient management^27,28^. Severity is moderate (~ 7), as frequent interruptions can compromise timely clinical interventions and patient monitoring effectiveness. Occurrence is medium (~ 5), related directly to the reliability and robustness of healthcare IT infrastructure, whereas detectability is often high (~ 4), readily identifiable through network monitoring systems and real-time technical alerts.

## Discussion

This study provides a structured and systematic approach to identifying, evaluating, and prioritizing risks in digital healthcare systems using FMEA. The analysis deliberately relied on lightweight, widely available tooling rather than custom software, making the workflow easily replicable across institutions with limited technical resources. The findings reveal that critical vulnerabilities are not uniformly distributed but vary significantly across different healthcare data categories and modalities. In clinical data, hazards such as unauthorized access and data corruption were identified as highly critical due to their direct implications for patient safety, diagnostic accuracy, and institutional trust. These insights align with growing concerns in the literature regarding the escalating cybersecurity risks in handling sensitive health information^20–24^. Similarly, operational data showed notable weaknesses related to delayed data entry and inadequate backup systems, highlighting the pressing need for real-time data synchronization, automated logging, and robust disaster recovery strategies^25,26,30–32^. Patient-reported data also posed unique challenges, with privacy breaches, data incompleteness, and demographic biases emerging as significant risks. These issues threaten the integrity of personalized care initiatives and reinforce prior research underscoring the need for rigorous governance in managing patient-generated health data^4,25,26,34–36,39^. Across data modalities, specific risk profiles were evident. Text and tabular data were prone to entry errors and inconsistencies, while image data faced issues like misprocessing and corruption. Audio and video data presented risks of degradation, transmission failures, and data loss, all of which directly impact the quality and reliability of remote care and telemedicine services.

These findings have critical implications for healthcare data management. Institutions must adopt robust access controls, such as multi-factor authentication and role-based permissions, to mitigate unauthorized access to sensitive data^45^. Furthermore, the implementation of automated validation tools and centralized data platforms can significantly enhance the accuracy and reliability of clinical and operational records while improving workflow efficiency^46–48^. In parallel, patient-reported data require enhanced protections through encryption and anonymization, as well as design interventions such as structured input forms and mandatory fields to ensure completeness and reduce bias^49,50^. Additionally, addressing risks in diverse data modalities also demands targeted strategies. Standardization through HL7 and FHIR frameworks can improve interoperability and reduce format inconsistencies^51^. The deployment of deduplication algorithms and expert oversight in AI-driven diagnostics can enhance processing accuracy, particularly for image and video data. In remote care contexts, maintaining multimedia quality through redundant infrastructure and real-time monitoring is essential for sustaining trust and clinical effectiveness^52^.

Looking forward, several strategic directions emerge. Intelligent data quality systems powered by AI can autonomously detect anomalies and correct inconsistencies, reducing clinician workload and improving data reliability^53^. Cybersecurity frameworks should incorporate encryption, behavioral analytics, and, where feasible, blockchain to secure health information with greater transparency and traceability^54^. To ensure fairness and trust in AI applications, inclusive training data, regular audits, and explainable AI are vital components^55^. Moreover, the growth of telemedicine requires further investment in transmission protocols and encryption standards to guarantee continuity of care and data confidentiality, especially in low-bandwidth settings^56^. Healthcare organizations must also adapt to evolving regulations such as HIPAA and GDPR through continuous audits and the adoption of global interoperability standards to facilitate secure cross-system data exchange^57^. Meanwhile, the integration of emerging technologies, such as wearables, IoT devices, and smart EHRs, offers promising avenues for improving real-time monitoring, predictive analytics, and data-driven decision-making^58,59^.

Furthermore, predictive modeling holds great potential for operational planning and proactive clinical interventions, provided it is supported by reliable, unbiased data and transparent validation processes^60^. In the realm of personalized medicine, success depends on the availability of standardized, high-quality, and bias-mitigated datasets that can support tailored treatment strategies based on individual characteristics^61^. By combining rigorous risk assessment with practical and forward-looking recommendations, this study contributes a novel and comprehensive framework for improving digital health data safety, quality, and resilience. The insights presented in this study are expected to aid healthcare providers, developers, and policymakers in developing secure, interoperable, and patient-centered digital health ecosystems.

To the end, this study makes four principal contributions to digital healthcare risk management. First, it is the first to operationalize FMEA across the full spectrum of healthcare data categories, including clinical, operational and patient-reported, and five major data modalities, such as text, image, tabular, audio, and video, showing that risk intensity is highly context-dependent rather than uniform. Second, through expert-validated scoring, it identifies and prioritizes critical data-related failure modes, such as unauthorized access, privacy breaches, data corruption, and delays in data entry, based on severity, occurrence, and detectability, thereby highlighting key vulnerabilities that threaten patient safety and system reliability. Third, it provides evidence-based mitigation strategies aligned with technical standards to address these high-priority risks, including enhanced access controls, automated data quality validation, improved interoperability using HL7/FHIR, and robust data backup mechanisms Finally, by integrating insights from academic literature, industry practices, and expert consultations, the study presents a structured and transferable framework for proactive risk assessment that can support safer and more efficient digital healthcare operations.

The framework and recommendations outlined here can directly benefit hospital risk managers, health IT vendors, policy makers, and patient advocacy groups by providing practical tools for safer, more resilient digital health infrastructures. Findings will be publicized through workshops with clinical stakeholders, open-access technical briefs for developers, and policy talks with regulatory bodies. Within the next 12 months, we aim to refine and test this approach in additional hospital departments, with a multicenter rollout and integration of automated scoring tools targeted within 18 months to enable continuous risk monitoring and validation.

## Data Availability

The data generated, used, and analyzed during the current study were available from the corresponding author upon reasonable request.
